# PANDAS have claws too: A case report of an adolescent female with concurrent PANDAS and Mood Disorder

**DOI:** 10.1192/j.eurpsy.2025.1259

**Published:** 2025-08-26

**Authors:** C. I. C. Uy, M. D. J. L. Calleja

**Affiliations:** 1 Department of Psychiatry, The Medical City, Pasig City, Philippines

## Abstract

**Introduction:**

Pediatric Autoimmune Neuropsychiatric Disorders Associated with Streptococcal Infection (PANDAS) is an autoimmune disease caused by Group A Streptococcus bacteria. It usually affects children 3 to 12 years of age, however, have also been reported among adolescents. PANDAS presents with neuropsychiatric symptoms which can overlap or coincide with existing psychiatric disorders that often emerge further as children get older. Identifying the causation of events may be challenging making such cases difficult to manage.

**Objectives:**

To understand the presentation of PANDAS with concurrent mood disorder and its management.

**Methods:**

Clinical case report

**Results:**

A 16-year-old female was initially admitted due to blank stares, disorganized behavior, paranoia, aggression, and depressive symptoms. Her EEG and MRI were normal. She did not tolerate Fluoxetine but improved with Olanzapine (12.5mg). At 17 years old, she had throat discomfort, colds, and headache with supportive management done. She felt depressed and suicidal due to school stress prompting her second admission. She was started on Escitalopram and Olanzapine was shifted to Quetiapine. Subsequently, she developed manic symptoms with hallucinations and worsened paranoia. Escitalopram was discontinued and Quetiapine was increased (300mg). Bipolar I Disorder with Psychotic Features was considered. Shortly after, she developed explosive motor and vocal tics, involuntary screeching with head gagging and jerking and urges to choke herself or hit her head on the wall. Quetiapine was discontinued and was placed on Clonazepam (0.25mg/8h). Work ups revealed normal EEG, positive ASO and ESR, and findings of Mild Rheumatic Heart Disease on 2D Echo, hence, the assessment of PANDAS. Treatment is multidisciplinary involving antimicrobial, immunomodulatory, and psychotherapeutic interventions. Medications were then adjusted to: Co-amoxiclav (625mg/10days) followed by Penicillin V (250mg/12h), Aripiprazole (15mg), and Divalproex Sodium (1000mg). Intravenous immunoglobulin was suggested due to her moderate to severe presentation. Henceforth, improvement of symptoms were noted and she was reintegrated back home.

**Conclusions:**

Diagnosing PANDAS can be complex among adolescent due to concurrent issues with similar symptom presentation. PANDAS is episodic in nature but may also have sawtooth-like presentation which may indicate first admission as an episode and the second, a flare up. The case could have also been a combination of PANDAS and Bipolar Disorder thus the severity. Regardless, the interplay of biological and psychological facets have evidently magnified her predispositions leading to an intense manifestation of symptoms.

**Disclosure of Interest:**

None Declared

